# Acute Exposure to Arsenic Affects Pupal Development and Neurological Functions in *Drosophila melanogaster*

**DOI:** 10.3390/toxics11040327

**Published:** 2023-03-30

**Authors:** Anushree, Md Zeeshan Ali, Anwar L. Bilgrami, Jawaid Ahsan

**Affiliations:** 1 *Drosophila* Behavior Laboratory, Department of Biotechnology, Central University of South Bihar, Gaya 824236, Bihar, India; 2 Deanship of Scientific Research, King Abdulaziz University, Jeddah 21589, Saudi Arabia

**Keywords:** arsenic, behavior, *Drosophila melanogaster*, LC50, learning, toxicity

## Abstract

Millions of people in developing countries are affected by arsenic (As) toxicity and its prevalence. Arsenic’s detrimental effects on humans have been amplified by an unacceptable level of exposure to food and drinking water, the ongoing rise in industrial usage, and several other occupational conditions. Due to increased cellular absorption and the ability to cross the blood–brain barrier (BBB), inorganic arsenic (iAs) is extremely hazardous to living organisms in its trivalent form. Arsenic toxicity damages an organism’s tissues and organs, resulting in skin cancer, circulatory system abnormalities, and central nervous system disorders. However, a competent model system is required to investigate the acute effects of arsenic on the brain, cognition ability, and to assess any behavioral impairment. Hence, *Drosophila*, with its short generation time, genomic similarities with humans, and its availability for robust behavioral paradigms, may be considered an ideal model for studying arsenic toxicity. The present study helps to understand the toxic effects of acute arsenic treatment on the behavior, cognition, and development of *Drosophila* in a time-dependent manner. We found that the exposure of fruit flies to arsenic significantly affected their locomotor abilities, pupae size, cognitive functions, and neurobehavioral impairment. Hence, providing a better understanding of how arsenic toxicity affects the brain leading to acute behavioral disorders and neurological alterations, this study will lead to a better understanding of the mechanisms.

## 1. Introduction

Arsenic originates from natural geologic sources due to various anthropogenic actions, such as mining, irrigational, agricultural, and industrial processes [1,2]. Food and contaminated water are primary sources of inorganic arsenic (iAs) exposure. Arsenic is naturally present in the groundwater in the south and southeast Asian regions, including India, Bangladesh, Nepal, Vietnam, and China [3,4]. Inorganic arsenic exists in two forms of oxidation, the trivalent and the pentavalent states. The trivalent form of arsenic is 60 times more toxic than the pentavalent form as it can cross the blood–brain barrier to enter the nervous system [5,6,7]. Its use in manufacturing paints, insecticides, wood preservatives, and pesticides has increased worldwide. According to the Agency for Toxic Substances and Disease Registry (ATSDR, 2013) [8], arsenic is ranked first on the priority list due to its increased usage in manufacturing pesticides. The World Health Organization (WHO, 2017) [9] listed arsenic as one of the ten most hazardous chemicals affecting human health. Acute and chronic toxicity due to the presence of arsenic in groundwater was reported to be a major public problem causing arsenicosis and cancer [10,11].

Arsenic (As) is a toxic metalloid, nearly odorless, tasteless, and it is ubiquitously present in nature. The acute effects of arsenic may be asymptomatic, but chronic exposure affects the organs and tissues; causing skin lesions and cancer, and leading to neurological impairment [12,13,14]. In various parts of the world, the concentration of arsenic has exceeded the maximum permitted exposure limit of 10 µg/L, as recommended by the WHO in 2017 [15,16,17]. The US National Research Council (NRC, 2000) [18] has reported that drinking water containing 50 mg/L may result in every 1 out of 100 cancer deaths. Arsenic was found to be as high as 1.35 mg/L in drinking water near oil depots in Aba, Nigeria [19]. Vegetables farmed in Bangladesh had 2–100-fold higher arsenic concentration than vegetables farmed in North America and the UK [20]. Arsenosugars and arsenobetaine were found in seaweeds and crustaceans, respectively [21]. The industrial arsenic used to produce antifungal wood preservatives further contaminates the soil. Arsenic levels in the blood of electronic trash-exposed individuals have been found to be 10 times greater than those of non-exposed individuals [22]. Smoking cigarettes and drinking water were linked to synergistic toxic effects caused by arsenic exposure [23]. The US Environmental Protection Agency (EPA) has estimated that approximately 0.04 to 0.09 µg of arsenic is inhaled by humans per day [24,25]. In cosmetics, a very high arsenic concentration of 11.1 mg per kilogram is reported in eye shadow [26]. When occupationally exposed to toxic arsenic, peripheral neuropathy, rash, or gastrointestinal symptoms are commonly observed [27].

Fewer studies are available on the toxic effects of arsenic on *Drosophila*. *Drosophila*, in its short life cycle of ten days includes four developmental stages: egg, larvae, pupae, and adults [28]. The mean life span of *Drosophila melanogaster* is 2–3 months. At 25 °C, the first instar larvae hatch out of eggs after one day, the next day they transform into second instar larvae, then in the following day, they enter the third instar larval stage which lasts for 2–3 days. Thus, the average length of the egg larval period is 5 to 6 days. Then, the third instar larvae pupate and the pupal life is about 4 days, followed by the emergence of adult flies. Thus at 25 °C, the life cycle is completed in about 10 days. Humans and fruit flies share some similarities, the genome of *Drosophila* conserves about 60 percent homology with human biological activity genes and approximately 75 percent similarity with human disease genes [29]. The fly offers a powerful alternative to investigate molecular aspects of toxicology and neurobehavior with its short life span, low-cost maintenance, and lack of ethical issues involved. Hence, *Drosophila* significantly accelerates functional research to discover various mechanisms of underlying diseases. Researchers have employed *Drosophila* as a useful genetic model to study responses toward odorants due to the presence of olfactory sensory neurons (OSNs) expressing odorant receptors (ORs) [30,31,32,33]. It has two pairs of olfactory organs, the antennae and the maxillary palps. Each antenna houses about 1200 OSNs and 410 olfactory sensilla, whereas the maxillary palp contains about 120 OSNs and 60 olfactory sensilla. [34]. Olfactory responses to ethyl acetate (EA), a volatile food odor and attractant are remarkably conserved across various strains of *Drosophila*.

The prevalence and industrial usage of inorganic arsenic (iAs) have become a public health concern worldwide, hence understanding its neurotoxic effects is of paramount importance. Epidemiological pieces of evidence from the human population indicate that iAs affect cognitive and intellectual functions of different developmental stages [35,36]. iAs is not yet proven to trigger neurodegeneration and mechanisms involving the neurotoxic effects of arsenic. There are toxicological and epidemiological evidence of arsenic toxicity in humans [37] and its potential role in increasing susceptibility to neurodegenerative disorders. Further research is needed to identify cellular and molecular mechanisms showing the effects of exposure to iAs on the development, neurotransmission, cognitive or behavioral functions, epigenetic regulation, and gene expression. Additionally, research on neurotoxicity due to aggregate exposure to iAs is still lacking. This research article focuses on the acute dose-dependent effects of arsenic neurotoxicity on the development, olfaction, cognition, locomotion, and learning behavior of *Drosophila melanogaster*.

## 2. Materials and Methods

### 2.1. Fly Husbandry, Breeding, and Collection of Flies

Wild-type flies of the Oregon R+ strain were reared on cornmeal media and maintained at 25 °C on a 12 h light/dark cycle in a BOD incubator. The media were comprised of high-grade 8 gm/L agar, 15 gm/L yeast extract, 80 gm/L corn, 20 gm/L dextrose, and 40 gm/L sucrose. Antifungal and antibacterial agents, such as 4 mL/L propionic acid and 0.6 mL/L orthophosphoric acid were added to the media after it was cooled down to room temperature. All of these chemicals were obtained from HiMedia (Mumbai, India). The flies were periodically transferred to fresh media bottles for proper breeding, growth, and health maintenance. The last pupal stage bottles were made fly-free and kept in the incubator. The bottles were manually inspected and it was confirmed that no fly was in the bottle. Following 18 h, newly emerged young flies were collected in fresh corn meal media vials. These collected flies were approximately of the same age. These flies were kept in those corn meal media vials for five days. These five-day old flies collected on the fifth day were used for all behavioral assays and experiments.

### 2.2. Chemicals

Sodium (meta) arsenite (NaAsO_2_) with ≥90% purity (MW 129.91 g/mol) was used for the treatment. The odorant, ethyl acetate (EA) was used in the behavior assays. Mineral oil was used as diluent (solvent). All of the highest grade chemicals were purchased from Sigma-Aldrich, St. Gallon, Switzerland.

#### Materials

The T-type polypropylene (PP) connector (Thermo Fisher Scientific, 6151–0500, Waltham, MA, USA) was used for olfactory behavior assays, a Vernier caliper (Zhart, Jaipur, India) with a measuring range between 0–150 mm (accuracy: ±0.02 mm/<0.001 in (<100 mm) ± 0.03 mm/0.001 in (>100–200 mm)) was used to measure the length and width of the pupae. A 50 mL glass measuring cylinder (Hirschmann, Germany) was used for the climbing (locomotory) assays.

### 2.3. Treatment and Estimation of the Lethal Concentration (LC50)

The polyethylene terephthalate (PET) bottles with a capacity of 50 mL obtained from the local market were used to treat the flies. A tissue bed was prepared at the base of each bottle. The surface of the tissue bed was made smooth and plain to avoid trapping flies within the tissue bed. A five percent sucrose solution was used as a solvent to prepare sodium arsenite at the concentrations of 0.5 mM, 1 mM, 1.5 mM, 2 mM, 3 mM, 4 mM, and 5 mM, as per the protocol [38]. For the control, only a 5 percent sucrose solution was used for all treatments. An equal volume (2 mL) of sodium arsenite solution (made in 5 percent sucrose solution) at specific concentrations was pipetted onto the tissue bed depending on the experiment. The soaked tissue bed with the solution was easy to feed the flies. Ten healthy flies (five individuals each of males and females) were transferred to each bottle, and their maximum survivability was studied. For determining LC50, the flies’ survivability was studied for 24 h at each arsenic concentration. For the statistical significance, three hundred flies were tested for the survival studies at each concentration with the total number of flies representing the results of 10 separate trials, each trial with 10 flies (five males and five females) and in triplicates. Statistically, LC50 represents the concentration of inorganic arsenic at which half of the flies’ population would die. The probit regression model was used to determine the LC50 of sodium arsenite in *Drosophila* within 24 h [39]. The *x*-axis and *y*-axis represent the log of concentrations of arsenic used for treating flies and the actual probability (probit, P) of dead flies at each concentration, respectively. For studying the acute effect of arsenic on the brain and neurobehavioral changes, a suitable arsenite concentration should be determined as having a toxic effect, but behavioral changes could also be analyzed within their tested survivability of 24 h. Based on the flies’ survivability, LC50, developmental, and neurobehavioral assays were performed with three concentrations of sodium arsenite, which were 1 mM, 1.5 mM, and 2 mM.

### 2.4. Morphological Changes in the Pupae

The development of eggs into pupae and alterations in their morphology were monitored in 100 mL PET bottles. Corn meal media containing 1 mM, 1.5 mM, or 2 mM sodium arsenite was poured into the PET bottles. The bottle with corn meal media without sodium arsenite served as the control. For egg laying, five individuals of five-day old virgin female flies and the same number of male flies were transferred into each bottle. Following 18 h of egg laying, all flies were removed from the bottles and the bottles were returned to the BOD incubator for the eggs to develop into pupae. The length and width of 120 pupae were measured, which developed in 10 different vials (from five experiments) with 1 mM arsenic media. The Vernier caliper was used to measure the length and width of the pupae that were developed in treated and control media bottles.

### 2.5. Behavioral Experimental Setup

#### 2.5.1. Climbing Assay

A negative geotaxis assay or climbing assay was used to study the effects of arsenic on the motor activity, such as climbing. A modified startle-induced negative geotaxis (SING), a frequently used paradigm, was used for determining locomotor ability in flies [40,41]. This assay describes how insects enclosed in a narrow column respond to a mild mechanical shock by ascending quickly upward. This experimental setup required a 50 mL glass cylinder (130 mm in length and 30 mm in diameter) for the fly to exhibit its climbing activity against gravity. Five-day old flies, treated with different concentrations of arsenic (1 mM, 1.5 mM, and 2 mM) for 18 h were tested for their climbing ability along with untreated control flies. The single fly was transferred into the glass measuring cylinder, and rested for 5 s before the experiment. The measuring cylinder was tapped at the base gently so that the fly remained at the bottom of the cylinder. The climbing activity of the fly was observed for 15 s. The experiment was repeated 40 times for each treated and untreated set. Twenty male and female individuals were tested individually for their locomotor ability. The distance climbed by the fly in 15 s was recorded by observing the distance (in mm) from the base of the cylinder to the mark on the paper strip. 

#### 2.5.2. Olfactory Two-Choice Assay

**Figure 1:**
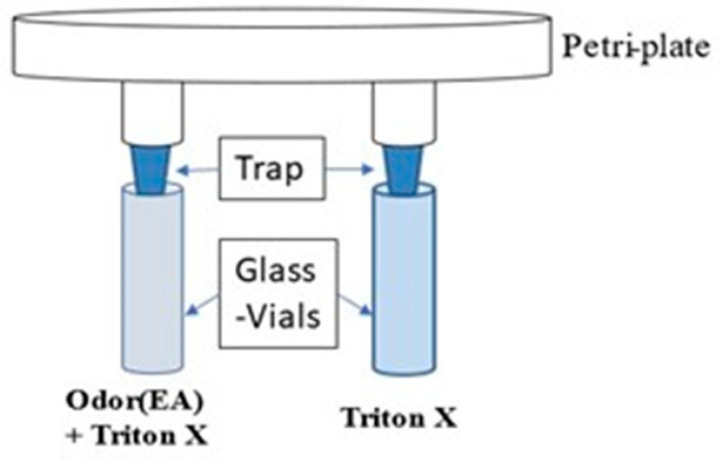
An illustration of an experimental two-choice olfactory assay designed for adult *Drosophila* flies. The two traps attached to the Petri plate do not allow flies to change their choice, once they enter the glass vial. One glass vial contained 300 µL of 0.2% Triton X-100 diluted in water (solvent), while the other contained 300 µL of ethyl acetate odor of 10^−2^ dilution in 0.2% Triton X-100.

This assay was adapted from Dekker et al., Reubenbauer et al., and Farhadian et al. [42,43,44], and modified to test five-day old flies for their olfaction and cognitive abilities. The experimental setup required a polypropylene (PP) Petri plate (90 mm), two glass vials of 10 mL each, and traps made from 1 mL microtip. Two traps were inserted into the Petri plate by making holes of specific dimensions (Figure 1), and they were made to enter into the two glass vials, thus connecting the Petri plate with the glass vials. The traps were positioned, such that the Petri plate’s lid fitted firmly, preventing flies from escaping, and allowing them to enter the glass vials through traps easily. Just before the experiment, one glass vial was pipetted with 300 µL of 0.2% Triton X-100 diluted in water as solvent, and in the other glass vial with 300 µL of ethyl acetate (EA) odor at 10^−2^ dilution in 0.2% Triton X-100 in water (solvent). The 10^−2^ dilution of EA was used as it is attractive to flies when used for extended testing durations. 

Earlier, the arsenic-treated flies (at 1 mM, 1.5 mM, and 2 mM) were starved for 4 h along with the control flies. The flies from each set were anesthetized on ice. Once they were anesthetized, forty flies (both males and females) from each set were transferred to different experimental Petri plates. The Petri plates were immediately covered with a lid. The setup with flies and glass vials (solvent and EA) was kept in complete darkness to avoid any kind of bias due to light. The experiment was repeated seven times in triplicates for each set of flies. Following 18 h, the number of flies remaining in the Petri plate and in each vial was counted for their preference between ethyl acetate and the solvent. 

The olfactory response index was calculated using the formula:

$$Olfactory\;response\;index\;I\left(RI\text{-}I\right)=\frac{O1-O2}{\left(O1+O2+P\right)}\times 100\%$$

where *O*1 = number of flies in the odor EA (10^−2^ dilution in 0.2% Triton X-100) vial

*O*2 = number of flies in the solvent (0.2% Triton X-100) vial

*P* = number of flies remaining in the Petri plate

The olfactory *RI-I* was multiplied by a hundred to determine the response in percentage. 

#### 2.5.3. Learning Assay

Training: The five-day old flies were trained in an associative appetitive learning paradigm modified from Schwaerzel et al. [45]. Three sets of flies were trained for the learning experiments: the control group, sucrose (SUC) group, and arsenic-treated group. The first set of untreated (no arsenic treatment) flies was trained with water associated with odor, representing the control group. The second set of untreated (no arsenic treatment) flies was trained with sucrose associated with odor, representing the sucrose group. The third and last set was of treated (arsenic-treated at specific concentrations) flies that were trained with sucrose associated with odor; it represented the arsenic-treated group of flies. All three sets of flies were starved for 4 h before training. In this appetitive learning paradigm, the flies were exposed for 2 min with ethyl acetate (EA) odor in association with sucrose (positive reinforcer or reward). For the training set up, a Whatman filter paper of specific dimensions was soaked overnight in 1 M sucrose solution and dried in air. The dried filter paper was rolled and placed in a vial to fit and cover completely. The lid placed on the top of the vial contained odor EA (10^−4^ dilution in mineral oil). Thirty flies (both males and females) were transferred to these vials for training in each experiment. The flies were exposed to either water/sucrose along with odor EA simultaneously for two minutes, for the association. The flies from all three sets were trained in similar ways. Following the training, the flies were tested for memory in an olfactory T-maze assay.

**Figure 2:**
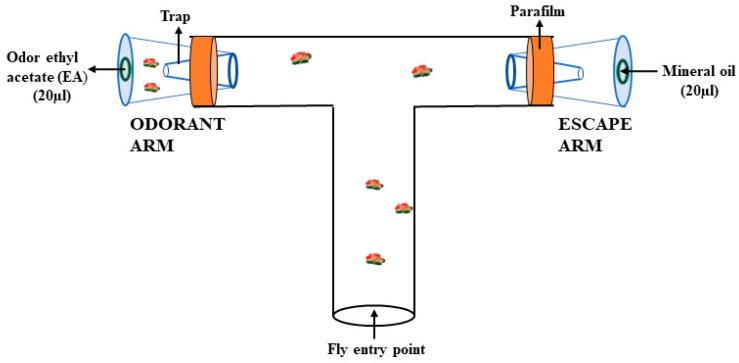
An illustration of an experimental T-maze testing assay designed for adult *Drosophila* flies. The two arms of the T-tube, the escape arm, and odorant arm are attached to the traps that do not allow the flies to change their choice, once they enter the trap. Parafilm helps in avoiding flies from being trapped in between the space and tight fitting of the trap with the T-tube. One trap contained 20 µL of mineral oil (solvent), while the other contained 20 µL of ethyl acetate odor of 10^−5^ dilution in mineral oil.

Testing in the T-maze assay: T-type connector tubes from Thermo Fisher Scientific were used for testing. A modified version of the T-maze testing assay was adapted from Chakraborty et al., Zamberlan et al., and Vittoria et al. [46,47,48]. Both arms of the T-tube were connected to a trap, as shown in Figure 2. The trained flies were immediately transferred to the T-maze. One arm (odor arm) of the T-maze contained odor EA (10^−5^ dilution in mineral oil), while the other arm (escape arm) had mineral oil as solvent/control. In the T-maze, two traps specifically made from micro tips were used at the end of two arms of the T-tube to trap the flies. A round Whatman filter paper disc was placed on the inner side of the cap of each trap. Twenty µL of odor EA (10^−5^ dilution in mineral oil) was pipetted onto a filter paper disc placed in the trap of one arm (odor arm), while 20 µL of mineral oil was pipetted on the other (escape arm). Immediately after the training of those thirty flies, they were transferred into the T-maze using a funnel, and a resting period of 30 s was given. Then, the movement of flies towards the odor arm or escape arm was monitored up to 30 min. The number of flies in two traps (in the odor arm and escape arm), response index, and learning index were determined after 30 min. Thirty trained flies were tested in the T-maze and the experiment was repeated seven times in triplicates for each sample. An average of 600 trained flies from each sample were tested for their learning. 

The response index II was calculated using the formula:

$$Response\;index\;II\left(RI\text{-}II\right)=\frac{No-Ne}{No+Ne}$$

where, *No* = number of flies in the odor arm of the T-maze

*Ne* = number of flies in the escape arm of the T-maze

The *RI-II* was multiplied by one hundred to determine the response in percentage. Relative learning index was measured for control, 1 mM, 1.5 mM, and 2 mM arsenic-treated flies. 

The formula used was:

$$Learning\;index\;relative\;to\;sucrose=\frac{{\left(RI\text{-}II\right)}_{trained\;untreated}-{\left(RI\text{-}II\right)}_{trained\;treated}}{{\left(RI\text{-}II\right)}_{trained\;untreated}}\times 100\%$$

### 2.6. Statistics

The statistical analysis was performed using GraphPad Prism (GraphPad Software, San Diego, CA, USA; version 8.0.2). The probit regression model was used for determining the LC50 value by analyzing the survivability of flies treated with different sodium arsenite concentrations for 24 h [38]. A Student *t*-test was applied to determine the significant differences between the samples. A one-way ANOVA was conducted to check the statistical significance between samples treated in different sodium arsenite concentrations. Significant *p* values were indicated as *** (≤0.001) or ** (≤0.01). The statistical significance was also analyzed by a Wilcoxon signed rank test with *p* < 0.0001. 

## 3. Results

### 3.1. Estimation of LC50

Sodium arsenite concentrations diluted in 5 percent sucrose solution were 1 mM, 1.5 mM, 2 mM, 3 mM, 4 mM, and 5 mM. Flies were able to survive for 18 h at the highest sodium arsenite concentrations of 5 mM. Survivability of the flies decreased from 4 days to 18 h with increasing arsenic concentrations from 0.5 mM to 5 mM, respectively. As compared to the control bottles, sodium arsenite treatment reduced the survivability of the flies in 1.5 mM and 2 mM concentrations by an average of 50 percent in 24 h. The flies surviving in arsenic-treated bottles were lethargic and sedentary. While in 1 mM and 0.5 mM bottles, only a few flies were observed to have any adverse effect on survivability after 24 h of arsenic treatment. Based on the flies’ survivability in the 0.5 mM arsenic treatment bottle, the duration was minimal for flies to exhibit any arsenic toxic impact within a day.

**Figure 3:**
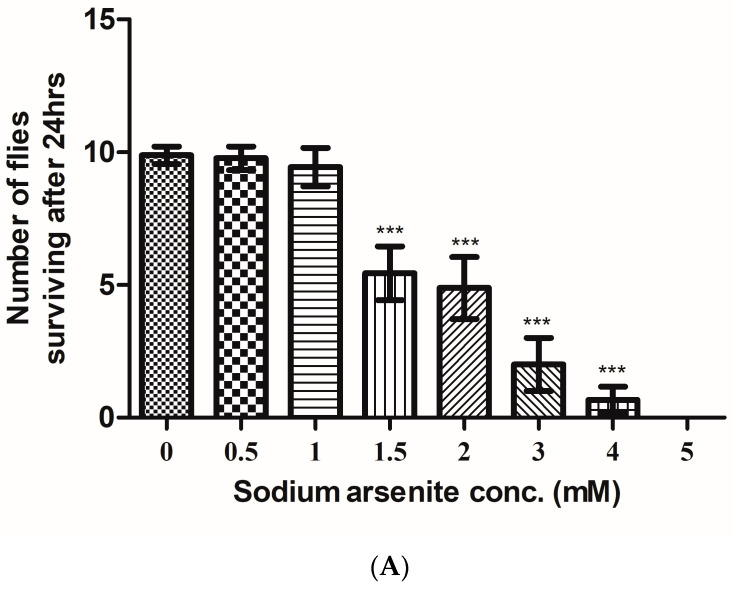

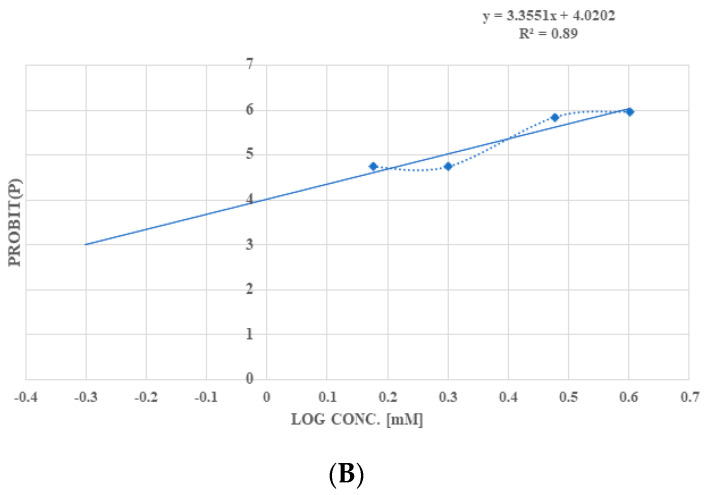
(**A**) The graph shows sodium arsenite (III) treated adult *Drosophila melanogaster* survivability within 24 h. Bars represent the means ± S.D. The significant mean difference (*p* < 0.05) between treated and untreated flies was analyzed by a one-way analysis of variance (ANOVA) (*** *p* < 0.0001). (**B**) The lethal concentration of sodium arsenite for flies within 24 h using the probit linear regression statistic is shown. Slope intercepts at 4.02 on the *y*-axis, log concentration is 0.29 mM, and the linear equation is y = 3.35x + 4.02 with R squared value as 0.89. The LC50 obtained is 1.96 mM.

Furthermore, 0.5 mM and 1 mM arsenite treated flies and control flies were monitored for seven days of survivability. Treated flies displayed toxic effects of arsenic on their survivability as compared to the control. The repeated trials showed the survivability of the flies inversely proportional to the sodium arsenite concentration within 24 h. The survivability of the lies decreased with the increase in sodium arsenite concentration. The average number of flies survived at different concentrations of sodium arsenite, i.e., 0.5 mM, 1 mM, 1.5 mM, 2 mM, 3 mM, 4 mM, and 5 mM were 9, 9, 5, 5, 1, 0, and 0, respectively (Figure 3A). 

The survivability of the flies at different concentrations of arsenite was statistically significant. According to the above data, the probit regression estimated a 1.96 mM concentration as LC50 of sodium arsenite for a sample size of 10 flies used for each experiment (Figure 3B). LC50 was given by the intercept of the best fit straight-line equation obtained as y = 3.35x + 4.02.

This linear regression equation establishes a relationship between sodium arsenite concentration and the number of flies dead within 24 h. The R-squared value was obtained as 0.89.

### 3.2. Pupae Size Measurement

First-instar larvae were hatched from the eggs only on corn meal containing lower concentrations of sodium arsenite and control media. Furthermore, there was a clear distinction in the later stages as pupae could be observed to develop only in 1 mM sodium arsenite containing media on the fifth day from the day eggs were laid. The first instar larvae were hatched from eggs on the fourth day in the control media bottle. At higher concentrations of arsenic (1.5 mM and 2 mM), no further stages of development were observed from day two. The pupa development was delayed in the arsenic-treated media as compared to the control media. The maximum and minimum lengths of the pupae in the control sets were measured as 3.52 mm and 3.1 mm, respectively.

**Figure 4:**
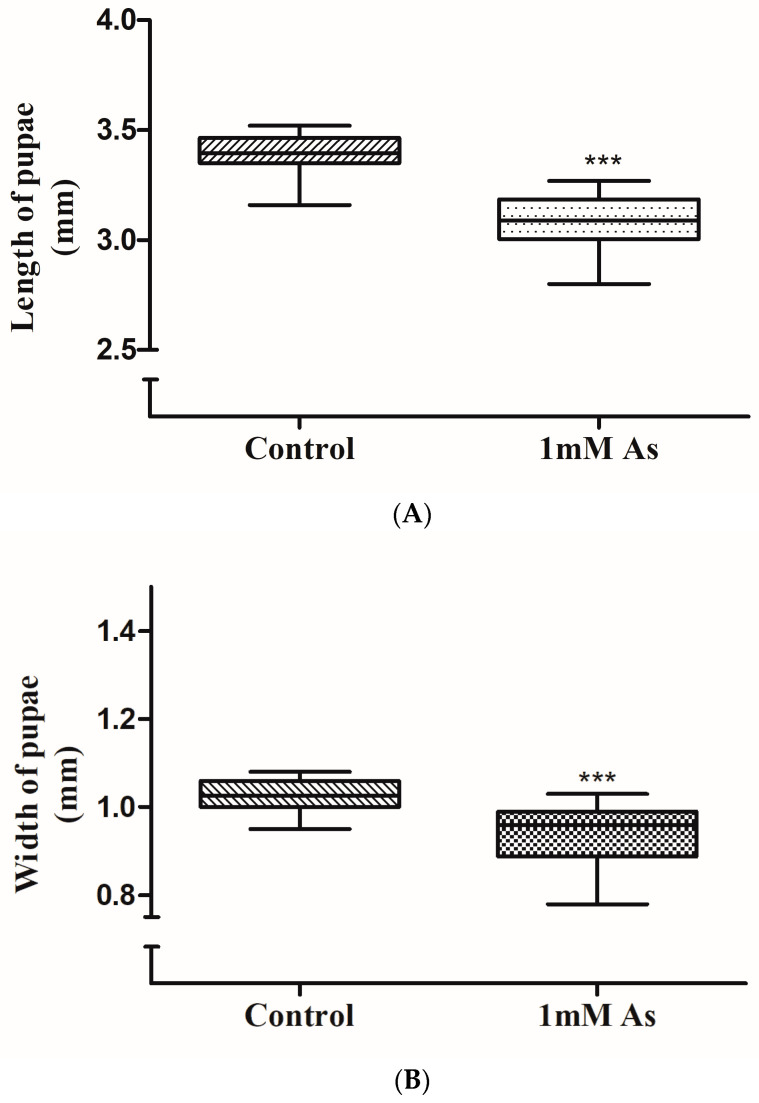
(**A**) The length of pupae that developed in 1 mM arsenite media and control media were measured and are represented. A decrease in the length (mm) of treated pupae compared to the control is apparent from the graph. The statistical significance (*p* < 0.05) was analyzed by the Wilcoxon signed rank test with *** *p* < 0.0001 and 95 per cent confidence interval. (**B**) The width of pupae that developed in 1 mM arsenite media and control media were measured and are represented. A decrease in the width (mm) of treated pupae compared to the control is apparent from the graph. The statistical significance (*p* < 0.05) was analyzed by the Wilcoxon signed rank test with *** *p* < 0.0001 and 95 per cent confidence interval.

The maximum and minimum lengths of arsenic-treated pupae were measured as 3.27 mm and 2.8 mm, respectively. The box plot (Figure 4A) represents the measurement of the length of pupae which were developed in the arsenic and control media. The maximum and minimum width of the pupae in the control bottle were measured as 1.08 mm and 0.95 mm, respectively. 

The maximum and minimum width of arsenic-treated pupae were measured as 1.03 mm and 0.78 mm, respectively. The box plot (Figure 4B) represented the measurement of the width of pupae which were developed in the arsenic and control media. The mean length of the control and treated pupae was 3.33 mm and 3.08 mm, respectively. While the mean width measured for the control and treated pupae was 1.02 mm and 0.93 mm. A minimal average relative decrease in the length and width of pupae developed in arsenic and control media were found. The relative decrease was 0.25 mm and 0.05 mm in length and width, respectively, and was statistically significant. Following measuring the length and width of the pupae, they were restored into their respective bottles to monitor their further development. The adult fly did not eclose from 1 mM arsenic media but flies eclosed from the control media bottle.

### 3.3. Climbing Assay

**Figure 5:**
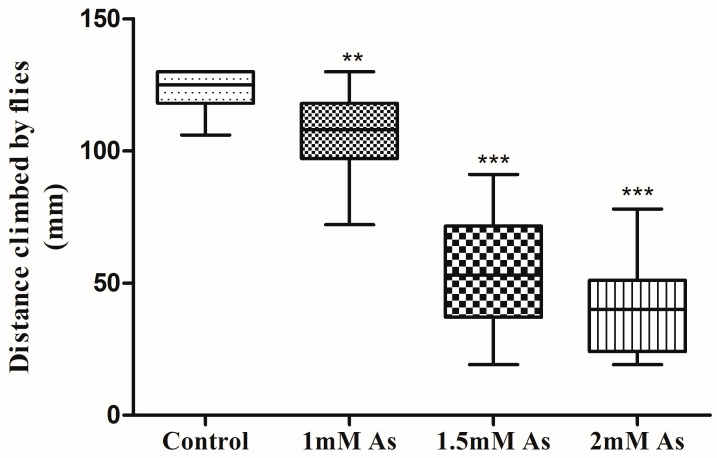
The box plot represents the negative geotaxis ability of adult flies treated at different concentrations of arsenite compared to the control flies. The distance is measured in millimeters (mm). The upper bar displays the maximum value from the dataset, while the lower bar displays the minimum value from the dataset. The significant mean difference (*p* < 0.05) between the untreated and treated climbing ability was analyzed by a one-way analysis of variance (ANOVA) (** *p* < 0.01; *** *p* < 0.001; R square = 0.712).

About 75 percent of control flies climbed the distance of 130 mm of the cylinder length, but the locomotor activity was not the same for the treated flies. The minimum and maximum distance climbed by control flies were 106 mm and 130 mm (Figure 5). 

The standard deviation and standard error of distance climbed by flies were 7.73 and 1.44, respectively. The 1 mM, 1.5 mM, and 2 mM arsenite-treated flies climbed up to an average distance of 107.5 mm, 55.31 mm, and 41.78 mm of the cylinder, respectively. The locomotor ability of flies treated at 1 mM, 1.5 mM, and 2 mM arsenic decreased to 12.6 percent, 55.03 percent, and 66 percent, respectively, compared to the control. In addition, flies treated with 2 mM arsenite exhibited minimal motor activity as compared to the control flies, and flies treated with 1 mM arsenite showed significant change in locomotion. The box plot (Figure 5) displays the locomotor and motor activity of three different sets of arsenic-treated flies compared to control flies.

### 3.4. Olfactory Two-Choice Assay

**Figure 6:**
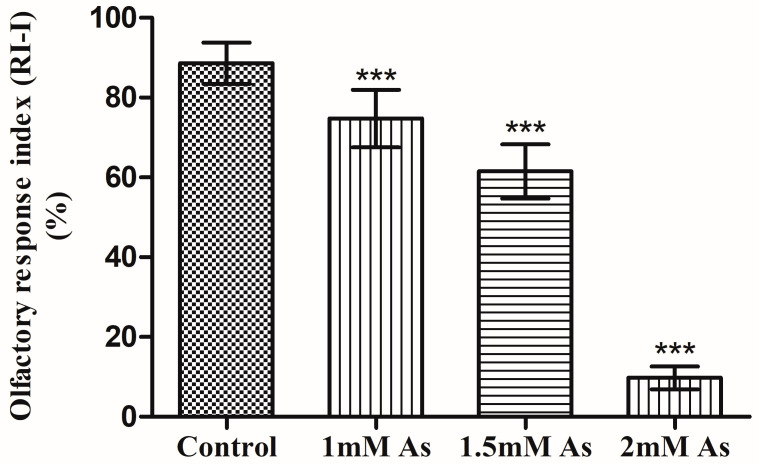
The graph represents an olfactory response index of control, 1 mM, 1.5 mM, and 2 mM arsenic-treated flies. The error bar represents the means ± S.D. The significant mean difference (*p* < 0.05) between RI-I mean of the different samples of flies were analyzed by a one-way analysis of variance (ANOVA) (*** *p* < 0.0001; R-squared = 0.771).

The average olfactory response index for the control flies was 88.62 percent. The arsenic-treated flies displayed different olfactory responses than the control flies. The average olfactory response of 1 mM arsenic-treated flies was 74.75 percent, which decreased by 13.88 ± 3.13 percent in comparison to the untreated flies (Figure 6). 

The olfactory response of 1.5 mM arsenic-treated flies was 61.5 percent, which was relatively decreased by about 27.13% ± 3.023 as compared to the untreated flies (Figure 6). Lastly, the olfactory response of 2 mM arsenic-treated flies was an average of 9%, which was very low, as only a small proportion of flies chose the vial, while most of the flies remained within the Petri plate. The olfactory response decreased as the concentration of sodium arsenite for treatment increased from 1 mM to 1.5 mM (Figure 6). An average difference of 13.25 percent in the olfactory response of 1.5 mM treated flies was found as compared to the 1 mM treated flies. There was a deleterious effect of arsenic on *D. melanogaster* olfaction at 2 mM arsenic concentration. 

### 3.5. Learning Assay: T-Maze Assay

**Figure 7:**
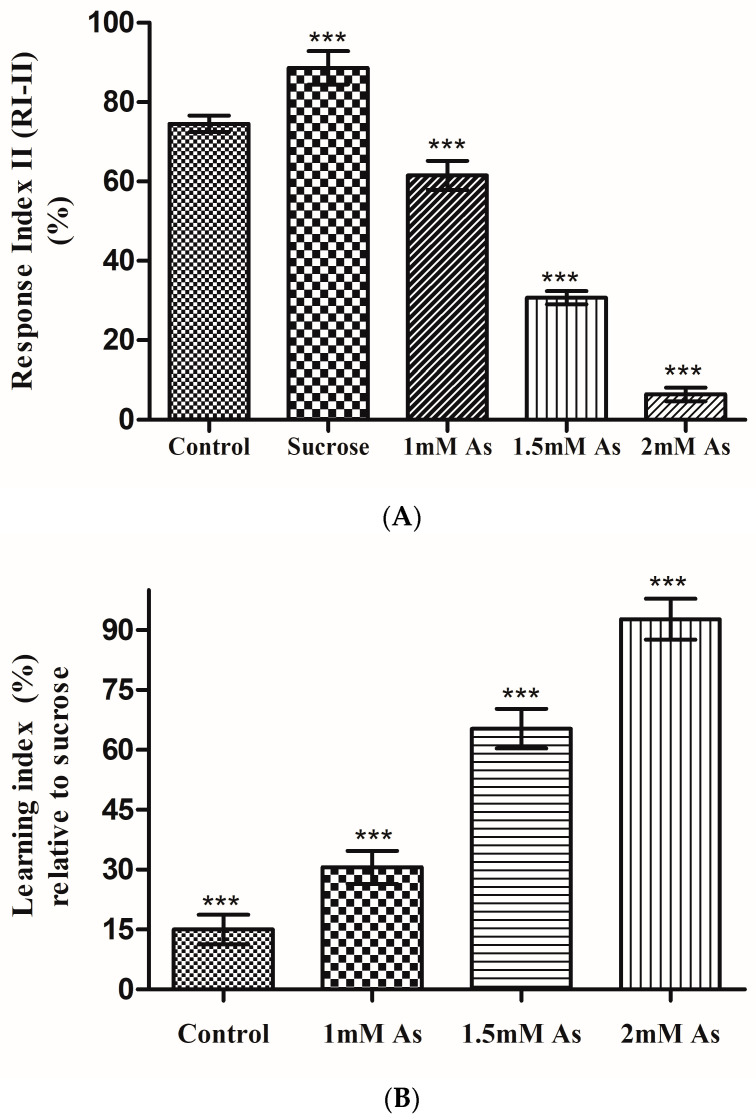
(**A**) The bar graph represents the response index-II of the control flies, sucrose untreated flies, treated flies at 1 mM As, 1.5 mM As, and 2 mM As. The error bar represents the means ± S.D. The significant mean difference (*p* < 0.05) between RI-II mean of the different sample of flies was analyzed by a one-way analysis of variance (ANOVA) (*** *p* < 0.0001; R squared = 0.974. (**B**) The bar graph represents the learning index of arsenic-treated flies and control flies relative to untreated sucrose flies, with the error bar representing the means ± S.D. The significant mean difference (*p* < 0.05) between the flies’ sample mean was analyzed by a one-way analysis of variance (ANOVA) (*** *p* < 0.0001).

The average response index (RI-II) of the control and untreated sucrose flies obtained was 75.33% and 88.64%, respectively. The average response index of seven repeated experimental trials of the control, untreated sucrose, and treated sucrose flies is represented graphically (Figure 7A). Each experimental trial was conducted in triplicate for data to be statistically significant and to eliminate any handling errors. The untreated sucrose flies displayed a maximum response and hence their learning was also at maximum as compared to the control and treated flies. Hence, the learning index of the control and arsenic-treated sucrose flies were estimated to be relative to the untreated sucrose flies, and are shown in a bar graph with an error bar representing the means ± S.D. (Figure 7B). Learning ability of flies was neither increased nor decreased when associated with the water. However, an average increase of 14.06% in the response of sucrose flies was observed, as they retained their learning of ethyl acetate odor associated with appetitive reinforcer sucrose, as compared to the control flies. The average response index of 1 mM, 1.5 mM, and 2 mM arsenic-treated flies was 61.52, 30.73, and 6.43%, respectively. The response of 1 mM arsenic-treated flies was decreased by an average of 13.81% and 27.11% as compared to control and sucrose flies, respectively. As the arsenic concentration for treatment increased, the fly’s response decreased. Hence, the maximum response was obtained by 1 mM arsenic-treated flies while the minimal response was obtained by 2 mM arsenic-treated flies.

Since untreated sucrose flies showed maximal response, the learning index was calculated for arsenic-treated flies relative to untreated sucrose flies. The average learning index of the control flies relative to the untreated sucrose flies was estimated to 15%. While the learning index of 1 mM, 1.5 mM, and 2 mM arsenic-treated flies relative to untreated sucrose was obtained as 30.59, 65.32, and 92.75%, respectively. The learning ability of 2 mM arsenic-treated flies was minimal in comparison to 1 mM arsenic-treated flies. So as the arsenic concentration was increased for the treatment, the learning index decreased evidently. There was an average decrease of 35 percent in the learning ability of 1 mM treated flies as compared to 1.5 mM treated flies. There was negligible learning when flies were treated at 2 mM arsenic concentration. In flies treated with 2 mM arsenite, no learning was observed as the average response (RI-II) of flies was only about 6%. The learning abilities of the control and arsenite-treated flies compared to untreated sucrose flies was less. 

## 4. Discussion

Human health has been threatened by acute and chronic exposure to arsenic, especially in developing countries, as it is highly toxic. Globally, people are affected by arsenic toxicity, which has become a major health threat causing gastrointestinal, respiratory, cardiovascular, genitourinary, endocrine, hematopoietic system, and skin diseases [49]. Studies have revealed that inorganic arsenic toxicity is also associated with neurological impairment in adult humans [50]. The toxic effects and disease implications of acute and chronic exposure to arsenic have been extensively studied. *Drosophila*, the principal model organism, has been used to study the neuro-toxicological effect of arsenic for its genome similarity with humans, easier culturing, and availability of robust behavioral paradigms.

Earlier studies demonstrated that inorganic arsenic species could not be converted into toxic derivatives within *Drosophila*, and it is not an ideal model to study in vivo genotoxicity and other toxic effects [38]. Nevertheless, the findings of the present study decipher that the neurotoxicity and behavioral anomalies occur due to arsenic in a dose-dependent manner. 

### 4.1. Effect of Arsenic on Survivability

Neurotoxicity and behavioral anomalies were responsible for attributing neuronal dysfunction and developmental effects on flies. There was almost no mortality of flies in the maximum number of repeated trials of the control bottles without arsenic. When the flies were fed from 0.5–5 mM acute concentration range of sodium arsenite, LC50 of inorganic arsenic for flies was estimated to be 1.96 mM in 24 h duration. In the present study, we found that increasing the dosage of arsenic had a detrimental effect on the life span of flies. There are conserved molecular pathways for heavy metal absorption and efflux, distribution, and regulatory systems in *D. melanogaster*, as well as in mammals [51]. Similar findings were observed by Bonilla-Ramirez et al. [52] in their study which discovered that the aberrant buildup of heavy metals in the flies reduces their life span. The poor regulation of heavy metals by flies may have resulted in the accumulation of arsenic in them, drastically shortening their lifespan. Our findings are similar to those of Rahman et al. [53] who reported that the presence of acute arsenic concentration in drinking water increases the mortality risk in young adults. Arsenic toxicity is possibly due to the generation of reactive oxygen species (ROS), which leads to increased oxidative stress, inflammation, and mitochondrial dysfunction [54]. The ROS generated further damage to the DNA and caused *Drosophila* death at higher concentrations. There have been reports of arsenic inducing superoxide dismutase (SOD) expression for a generation of O_2_ accompanying the enhanced activity of enzyme heme oxygenase to produce reactive ions [55]. Furthermore, evidence suggests that arsenic might stimulate cell signaling and trigger transcription factors and NADH oxidase to enhance the production of H_2_O_2_ and O^2−^ [56,57,58]. Multiple mechanisms might be involved in arsenic-induced DNA damage, but H_2_O_2_ and O_2_ have been the primary ROS involved in arsenic-induced DNA damage. However, a detailed exploration of epigenetic signatures and metabolic changes (bioenergetics, redox, neurotransmitters) is still needed, which have the potential to be used in disease mechanisms as biomarkers.

### 4.2. Effect of Arsenic on Development and Growth

Arsenic has been widely known as a teratogen that can affect the growth and development of a fetus. Concentrations and duration of arsenic play a significant role in influencing retardation in the growth and death of the fetus [59,60]. Studies related to the effect of arsenic toxicity on the development of flies in a time-dependent manner were reported earlier [61]. Several studies have found that an increase in fetal and infant mortality or impaired fetal growth might be due to arsenic exposure during development, since arsenic has the potential to move across membranes quickly [62]. The developmental stages of flies were significantly affected by the presence of sodium arsenite in the media. In other words, there is an apparent link between arsenic toxicity, internal dose (body burden), and pupation. Larvae must attain a sufficient size before pupating, and hormonal signaling is implemented to trigger pupal transition [63,64]. There are several highly conserved developmental pathways in flies that control both processes of pupariation and signaling pathway that are significant, such as the insulin-like peptide (Dilp8-Lgr3) pathway that regulates overall growth [63] and pupariation. This also involves the ecdysone hormonal pathway [64]. Larval size and hormonal signaling are crucial for larvae to develop into pupae, that are evidently affected due to arsenic toxicity. The average lengths and width of pupae developed in arsenic media were smaller than the control. A minimal but significant difference in length and width of 0.25 mm and 0.05 mm in arsenic-treated pupae as compared to control media pupae was observed in this study. An average decrease of 7.5 percent in length and 5 percent in width of pupae were found when developed in the presence of arsenic in media as compared to control media. The results of the present study correspond with those from the developmental study by Polak et al. [65] on flies indicating fluctuating symmetry due to arsenic toxicity. One of the studies on other animal models showed a more significant percentage decrease in tiny muscle fibers along with low birth weight due to arsenic [66]. Even human epidemiological studies reported a reduction in the birth weight of babies whose mothers were exposed to arsenic in drinking water [67,68]. Reports suggest that during development, arsenic exposure can affect neuronal progenitor cell development, differentiation, and functioning [69], which might also be responsible for the morphological changes.

### 4.3. Effect of Arsenic on the Locomotor Ability

Locomotion is an integral behavior of most animals, influenced by age, sex, genetics, and environmental factors [70]. Innate negative geotaxis response in *Drosophila* requires an intact sensory, interneuron, and motoneuron circuity. There is a complex association between the neural circuits and the motor system. Furthermore, the *Drosophila* nervous system uses dopamine neurotransmitters to control locomotion, just as it does in vertebrates. In our study, the average distance climbed by arsenic-treated flies at 2 mM and 1 mM decreased by a maximum of 33.9 percent and a minimum of 3.89 percent, respectively. In the flies treated at 1.5 mM arsenic concentration, locomotor ability decreased by 23.39 percent as compared to the control flies. Thus, as arsenic concentration increased, the locomotor ability in *Drosophila* decreased as compared to the control. Hence, it is evident from this study that there is an impairment in the climbing ability of the flies due to arsenic toxicity, which might be either due to genetic manipulation or interneuron circuitry of the central nervous system. The findings of environmental toxins and heavy metals indiscriminately diminishing dopaminergic neuron clusters in flies [71,72] and the human brain [73,74], play an essential role in their locomotion. 

Interestingly, the Parkinson’s disease-linked human alpha-synuclein gene expression results in a similar negative geotaxis locomotor dysfunction in flies [75,76]. The locomotor deficit in flies might further enumerate an essential clinical feature of Parkinson’s disease, which can be investigated further using cellular and molecular techniques. Furthermore, the climbing and motor activities were impaired in many *Drosophila* models of human neurological disorders, such as Huntington’s disease, Parkinson’s disease, and spinocerebellar ataxia [77], indicating the possibility of a similar mechanism in arsenic-inducing neurological disorders.

### 4.4. Effect of Arsenic on the Cognitive Response of Olfaction

Arsenic affects nearly every cellular process and organ function [78]. *Drosophila*, a standard olfactory system model, provides an excellent opportunity to examine a lack of sensing and induction signals that impact the health of sensory neurons [79,80,81,82]. Earlier studies of acute arsenic toxicity by Muñiz Ortiz et al. [83] on transgenic *Drosophila* demonstrated effects of the methylation of arsenic into a +3-oxidation state on organismal longevity. However, there was no mention of the adverse effects of arsenic on chromosomal integrity related to olfaction and cognitive responses which was investigated in our study. The olfactory response index (RI) estimated in the present study using the two-choice behavioral assay exhibits the effect of arsenic on olfaction and cognitive response. In this study, the average percentage of olfactory RI estimated for arsenic-treated flies was a maximum of 75 percent and a minimum of no response at a higher arsenic concentration. About 88 percent of the control flies responded to the ethyl acetate (EA) odor. Relative olfactory responses decreased between 15.6 and 30.6 percent depending on arsenic concentration as compared to the control flies. The decrease in olfactory response became more pronounced with increasing concentrations of arsenic, and even a complete ablation of the *Drosophila* olfactory response to ethyl acetate was observed at higher arsenic concentrations. The decrease in olfactory sensing ability reflects that there might be a strong possibility of olfaction-dependent sensory neuron impairment. The dysfunctioning of olfaction in *Drosophila* in the present study corresponds with the acute arsenic exposure (1000 ppm for 3–10 days) in swine caused demyelination of peripheral nerve fibers, axonal damages, ataxia [83], and neuronal myelin loss or abnormal myelination causing severe motor and sensory dysfunctions in the brain [84]. The neuronal myelin loss or abnormal myelination leads to severe motor and sensory dysfunctions in the brain [85]. In this study, the inability of the flies to sense ethyl acetate odor with increasing arsenic concentration could be due to neuronal impairments. Olfactory sensory neurons affected by arsenic may be amenable to genetic screens to understand the molecular mechanism of arsenic-dependent sensory degeneration. Various physiological conditions, including aging and neurodegenerative diseases, cause cognitive decline that impairs decision-making [86], which might also be one of the significant implications of arsenic-related olfactory deficit. 

### 4.5. Effect of Arsenic on the Neurological Ability of Learning

The ability of arsenic to cross the blood–brain barrier (BBB) makes it hazardous by potentially affecting the central nervous system [87]. Several pathways reported to be involved in learning, memory, movement, and decision-making are affected by arsenic, which is similar to the findings in our study [88,89]. In another study, arsenic-induced neurotoxicity was concluded to occur primarily due to oxidative stress for their incapable neuron signals to detoxify reactive oxygen species (ROS) and glutathione production [90]. The present study estimated that the learning ability of odor in flies associated with appetitive positive reinforcers was enhanced by 18.85 percent as compared to without sucrose (appetitive reinforcer)-associated odor. While the sucrose-associated learning ability of arsenic-treated flies decreased as the concentration of arsenic was increased. The relative learning ability of arsenic-treated flies observed a maximum of 65.4 percent learning index and a minimum of 30.5 percent decrease depending on arsenic concentration compared to non-treated flies. Hence, the data indicates that arsenic has a significant role in impairing neurological learning ability. Oxidative stress and increased levels of ROS caused due to arsenic exposure possibly affected the downregulation of antioxidant enzymes and neurodegenerative changes by aggregation of proteins, such as Tau, Aβ, or α-syn. Therefore, it might further affect the neurological ability to learn in arsenic-treated *Drosophila* flies. Moreover, these probable neuronal functional disturbances might be associated with several neurodegenerative diseases. Since biomarkers are not available for neurodegenerative diseases, such as Alzheimer’s or Parkinson’s for early diagnosis and timely treatment, the arsenic-treated *Drosophila* could be a valuable source for studying biomarkers and genetic factor manipulations. 

## 5. Conclusions

Our findings in this study reveal that acute exposure to different concentrations of arsenic significantly reduces the life span of flies within 24 h in a dose-dependent manner. Furthermore, acute arsenic effects lead to dose-dependent mortality, impaired growth, morphology, and developmental defects in *D. melanogaster*. Furthermore, we found that exposure to arsenic also affects behavior and other cognitive functions due to heavy metal homeostasis impairment in the fly. In addition, an association between arsenic exposure and neurodegeneration could be further investigated at different levels for biomarkers and drug development. However, it is uncertain, how exactly arsenic acts and the precise mechanism by which arsenic may lead to neurological impairment or neurodegeneration. Earlier studies on the effect of arsenic on flies [38,61,65,83] were mainly focused on development and toxicity. Previously exposure to arsenic-affected cognition in *Drosophila* larvae was published but not in adult flies [91]. To our knowledge, this is the first study on the neurotoxic effects of arsenic on cognition and behavior in adult flies. Further research is required to understand how heavy metals induce functional impairment in neurons or neuronal death in *Drosophila.* Therefore, our studies establish *D. melanogaster* as a valuable and versatile non-mammalian model system for a deeper understanding of human arsenic neurotoxicity and associated neurological diseases, including post-exposure behavioral impairment.

## Data Availability

The data presented in this study are available within the article. Additional data could be requested from the corresponding author.

## References

[1] Sarkar A., Paul B. (2017). The global menace of Arsenic and its conventional remediation—A critical review. Chemosphere.

[2] Zheng Y. (2017). Lessons Learned from Arsenic Mitigation among Private Well Households. Curr. Environ. Health Rep..

[3] Ravenscroft P., Brammer H., Richards K.S. (2009). Arsenic Pollution: A Global Synthesis.

[4] McArthur J.M., Sikdar P.K. (2019). Arsenic in groundwater. Groundwater Development and Management.

[5] Liu Z., Shen J., Carbrey J.M., Mukhopadhyay R., Agre P., Rosen B.P. (2002). Arsenite transport by mammalian aquaglyceroporins AQP7 and AQP9. Proc. Natl. Acad. Sci. USA.

[6] Torres-Avila M., Leal-Galicia P., Sánchez-Peña L.C. (2010). Arsenite induces aquaglyceroporin 9 expression in murine livers. Environ. Res..

[7] Calatayud M., Barrios J.A., Vélez D., Devesa V. (2012). In vitro study of transporters involved in intestinal absorption of inorganic Arsenic. Chem. Res. Toxicol..

[8] ATSDR—Agency for Toxic Substances and Disease Registry (2013). CAS ID#: 7440-38-2. http://www.atsdr.cdc.gov/substances/toxsubstance.aspx?toxid=3.

[9] WHO (2017). Health Impacts of Chemicals: Arsenic. http://www.who.int/ipcs/assessment/public_health/Arsenic/en/.

[10] Ahmad S.A., Khan M.H., Haque M. (2018). Arsenic contamination in groundwater in Bangladesh: Implications and challenges for healthcare policy. Risk Manag. Healthc. Policy.

[11] Baker B.A., Cassano V.A., Murray C., ACOEM Task Force on Arsenic Exposure (2018). Arsenic Exposure, Assessment, Toxicity, Diagnosis, and Management: Guidance for Occupational and Environmental Physicians. J. Occup. Environ. Med..

[12] Hong Y.S., Ye B.J., Kim Y.M., Kim B.G., Kang G.H., Kim J.J., Song K.H., Kim Y.H., Seo J.W. (2017). Investigation of Health Effects According to the Exposure of Low Concentration Arsenic Contaminated Ground Water. Int. J. Environ. Res. Public Health.

[13] Tsuji J.S., Chang E.T., Gentry P.R., Clewell H.J., Boffetta P., Cohen S.M. (2019). Dose-response for assessing the cancer risk of inorganic Arsenic in drinking water: The scientific basis for use of a threshold approach. Crit. Rev. Toxicol..

[14] Palma-Lara I., Martínez-Castillo M., Quintana-Pérez J.C. (2010). Arsenic exposure: A public health problem leading to several cancers. Regul. Toxicol. Pharmacol..

[15] Rahman M.A., Rahman A., Khan M., Renzaho A. (2018). Human health risks and socio-economic perspectives of Arsenic exposure in Bangladesh: A scoping review. Ecotoxicol. Environ. Saf..

[16] Rahman A., Rahaman H. (2018). Contamination of Arsenic, manganese and coliform bacteria in groundwater at Kushtia District, Bangladesh: Human health vulnerabilities. J. Water Health.

[17] Ali W., Rasool A., Junaid M., Zhang H. (2019). A comprehensive review on current status, mechanism, and possible sources of Arsenic contamination in groundwater: A global perspective with prominence of Pakistan scenario. Environ. Geochem. Health.

[18] NRC (National Research Council), U.S. Subcommittee on Arsenic in Drinking Water (2000). Arsenic in Drinking Water.

[19] Akakuru O.C., Akudinobi B.E.B., Usman A.O. (2017). Organic and heavy metal assessment of groundwater sources around Nigeria national petroleum cooperation oil depot Aba, South-Eastern Nigeria. J. Nat. Sci. Res..

[20] Le X.C., Cullen W.R., Reimer K.J. (1994). Human urinary Arsenic excretion after one-time ingestion of seaweed, crab, and shrimp. Clin. Chem..

[21] Lu F.J. (1990). Blackfoot disease: Arsenic or humic acid?. Lancet.

[22] Igharo G.O., Anetor J.I., Osibanjo O.O., Osadolor H.B., Dike K.C. (2014). Toxic metal levels in Nigerian electronic waste workers indicate occupational metal toxicity associated with crude electronic waste management practices. Biokemistry.

[23] Morales K.H., Ryan L., Kuo T.L., Wu M.M., Chen C.J. (2000). Risk of internal cancers from Arsenic in drinking water. Environ. Health Perspect..

[24] Sahu R., Saxena P., Johnson S., Mathur H., Agarwal H. (2014). Heavy Metals in Cosmetics. Centre for Science and Environment. https://cdn.cseindia.org/userfiles/Heavy_Metals_in_Cosmetics_Report.pdf.

[25] Yadav M.K., Saidulu D., Gupta A.K., Ghosal P.S., Mukherjee A. (2021). Status and management of Arsenic pollution in groundwater: A comprehensive appraisal of recent global scenario, human health impacts, sustainable field-scale treatment technologies. J. Environ. Chem. Eng..

[26] Saint-Jacques N., Parker L., Brown P., Dummer T.J. (2014). Arsenic in drinking water and urinary tract cancers: A systematic review of 30 years of epidemiological evidence. Environ. Health Glob. Access Sci. Source.

[27] Beckett W.S., Moore J.L., Keogh J.P., Bleecker M.L. (1986). Acute encephalopathy due to occupational exposure to Arsenic. Br. J. Ind. Med..

[28] Johnson A.A., Stolzing A. (2019). The role of lipid metabolism in aging, lifespan regulation, and age-related disease. Aging Cell.

[29] Ugur B., Chen K., Bellen H.J. (2016). *Drosophila* tools and assays for the study of human diseases. Dis. Model. Mech..

[30] Buck L., Axel R. (1991). A novel multigene family may encode odorant receptors: A molecular basis for odor recognition. Cell.

[31] Chess A., Buck L., Dowling M.M., Axel R., Ngai J. (1992). Molecular biology of smell: Expression of the multigene family encoding putative odorant receptors. Cold Spring Harb. Symp. Quant. Biol..

[32] Vosshall L.B., Wong A.M., Axel R. (2000). An olfactory sensory map in the fly brain. Cell.

[33] Carlson J.R. (2001). Functional expression of a *Drosophila* odor receptor. Proc. Natl. Acad. Sci. USA.

[34] Laissue P.P., Vosshall L.B. (2008). The olfactory sensory map in *Drosophila*. Adv. Exp. Med. Biol..

[35] Tolins M., Ruchirawat M., Landrigan P. (2014). The developmental neurotoxicity of Arsenic: Cognitive and behavioral consequences of early life exposure. Ann. Glob. Health.

[36] Garza-Lombó C., Pappa A., Panayiotidis M.I., Gonsebatt M.E., Franco R. (2019). Arsenic-induced neurotoxicity: A mechanistic appraisal. J. Biol. Inorg. Chem..

[37] Carlin D.J., Naujokas M.F., Bradham K.D., Cowden J., Heacock M., Henry H.F., Lee J.S., Thomas D.J., Thompson C., Tokar E.J. (2016). Arsenic and Environmental Health: State of the Science and Future Research Opportunities. Environ. Health Perspect..

[38] Rizki M., Kossatz E., Velázquez A., Creus A., Farina M., Fortaner S., Sabbioni E., Marcos R. (2006). Metabolism of Arsenic in *Drosophila melanogaster* and the genotoxicity of dimethylarsinic acid in the Drosophila wing spot test. Environ. Mol. Mutagen.

[39] Finney D.J. (1952). Probit analysis; a statistical treatment of the sigmoid response curve. J. R. Stat. Soc..

[40] Sun J., Xu A.Q., Giraud J., Poppinga H., Riemensperger T., Fiala A., Birman S. (2018). Neural Control of Startle-Induced Locomotion by the Mushroom Bodies and Associated Neurons in *Drosophila*. Front. Syst. Neurosci..

[41] Klimaczewski C.V., Ecker A., Piccoli B., Aschner M., Barbosa N.V., Rocha J.B.T. (2018). Peumus boldus attenuates copper-induced toxicity in *Drosophila melanogaster*. Biomed. Pharmacother. Biomed. Pharmacother..

[42] Dekker T., Ibba I., Siju K.P., Stensmyr M.C., Hansson B.S. (2006). Olfactory shifts parallel superspecialism for toxic fruit in *Drosophila melanogaster* sibling, *D. sechellia*. Curr. Biol..

[43] Ruebenbauer A., Schlyter F., Hansson B.S., Löfstedt C., Larsson M.C. (2008). Genetic variability and robustness of host odor preference in *Drosophila melanogaster*. Curr. Biol..

[44] Farhadian S.F., Suárez-Fariñas M., Cho C.E., Pellegrino M., Vosshall L.B. (2012). Post-fasting olfactory, transcriptional, and feeding responses in *Drosophila*. Physiol. Behav..

[45] Schwaerzel M., Monastirioti M., Scholz H., Friggi-Grelin F., Birman S., Heisenberg M. (2003). Dopamine and octopamine differentiate between aversive and appetitive olfactory memories in *Drosophila*. J. Neurosci. Off. J. Soc. Neurosci..

[46] Chakraborty T.S., Goswami S.P., Siddiqi O. (2009). Sensory correlates of imaginal conditioning in *Drosophila melanogaster*. J. Neurogenet..

[47] Zamberlan D.C., Halmenschelager P.T., Silva L.F.O., da Rocha J.B.T. (2020). Copper decreases associative learning and memory in *Drosophila melanogaster*. Sci. Total Environ..

[48] Vittoria M., Tilmann A., Claudia B., Alexandros K. (2020). Kanellopoulos, Modelling Learning and Memory in *Drosophila* to Understand Intellectual Disabilities. Neuroscience.

[49] Duker A.A., Carranza E.J., Hale M. (2005). Arsenic geochemistry and health. Environ. Int..

[50] Rodríguez V.M., Jiménez-Capdeville M.E., Giordano M. (2003). The effects of Arsenic exposure on the nervous system. Toxicol. Lett..

[51] Bahadorani S., Bahadorani P., Marcon E., Walker D.W., Hilliker A.J.A. (2010). *Drosophila* model of Menkes disease reveals a role for DmATP7 in copper absorption and neurodevelopment. Dis. Models Mech..

[52] Bonilla-Ramirez L., Jimenez-Del-Rio M., Velez-Pardo C. (2011). Acute and chronic metal exposure impairs locomotion activity in *Drosophila melanogaster*: A model to study Parkinsonism. Biometals.

[53] Rahman M., Sohel N., Yunus F.M., Alam N., Nahar Q., Streatfield P.K., Yunus M. (2019). Arsenic exposure and young adult’s mortality risk: A 13-year follow-up study in Matlab, Bangladesh. Environ. Int..

[54] Niño S.A., Martel-Gallegos G., Castro-Zavala A., Ortega-Berlanga B., Delgado J.M., Hernández-Mendoza H., Romero-Guzmán E., Ríos-Lugo J., Rosales-Mendoza S., Jiménez-Capdeville M.E. (2018). Chronic Arsenic Exposure Increases Aβ_(1-42)_ Production and Receptor for Advanced GlyCation End Products Expression in Rat Brain. Chem. Res. Toxicol..

[55] Lee T.C., Ho I.C. (1995). Modulation of cellular antioxidant defense activities by sodium arsenite in human fibroblasts. Arch. Toxicol..

[56] Chen Y.C., Lin-Shiau S.Y., Lin J.K. (1998). Involvement of reactive oxygen species and caspase 3 activation in arsenite-induced apoptosis. J. Cell. Physiol..

[57] Barchowsky A., Klei L.R., Dudek E.J., Swartz H.M., James P.E. (1999). Stimulation of reactive oxygen, but not reactive nitrogen species, in vascular endothelial cells exposed to low levels of arsenite. Free Radic. Biol. Med..

[58] Lynn S., Gurr J.R., Lai H.T., Jan K.Y. (2000). NADH oxidase activation is involved in arsenite-induced oxidative DNA damage in human vascular smooth muscle cells. Circ. Res..

[59] Tabacova S., Baird D.D., Balabaeva L., Lolova D., Petrov I. (1994). Placental Arsenic and cadmium in relation to lipid peroxides and glutathione levels in maternal-infant pairs from a copper smelter area. Placenta.

[60] Golub M.S., Macintosh M.S., Baumrind N. (1998). Developmental and reproductive toxicity of inorganic Arsenic: Animal studies and human concerns. J. Toxicol. Environ. Health Part B Crit. Rev..

[61] Beamish C.R., Love T.M., Rand M.D. (2021). Developmental Toxicology of Metal Mixtures in *Drosophila*: Unique Properties of Potency and Interactions of Mercury Isoforms. Int. J. Mol. Sci..

[62] Marie V. (2009). Effects of Arsenic on maternal and fetal health. Annu. Rev. Nutr..

[63] Jaszczak J.S., Wolpe J.B., Bhandari R., Jaszczak R.G., Halme A. (2016). Growth Coordination During *Drosophila melanogaster* Imaginal Disc Regeneration Is Mediated by Signaling Through the Relaxin Receptor Lgr3 in the Prothoracic Gland. Genetics.

[64] Riddiford L.M. (1993). Hormone receptors and the regulation of insect metamorphosis. Receptor.

[65] Polak M., Opoka R., Cartwright I.L. (2002). Response of fluctuating asymmetry to Arsenic toxicity: Support for the developmental selection hypothesis. Environ. Pollut..

[66] Handel S.E., Stickland N.C. (1987). The growth and differentiation of porcine skeletal muscle fibre types and the influence of birthweight. J. Anat..

[67] Hopenhayn C., Ferreccio C., Browning S.R., Huang B., Peralta C., Gibb H., Hertz-Picciotto I. (2003). Arsenic exposure from drinking water and birth weight. Epidemiology.

[68] Rahman A., Vahter M., Smith A.H., Nermell B., Yunus M., El Arifeen S., Persson L.A., Ekström E.C. (2009). Arsenic exposure during pregnancy and size at birth: A prospective cohort study in Bangladesh. Am. J. Epidemiol..

[69] McMichael B.D., Perego M.C., Darling C.L. (2021). Long-term Arsenic exposure impairs differentiation in mouse embryonal stem cells. J. Appl. Toxicol..

[70] Woods J.K., Kowalski S., Rogina B. (2014). Determination of the spontaneous locomotor activity in *Drosophila melanogaster*. J. Vis. Exp..

[71] Coulom H., Birman S. (2004). Chronic exposure to rotenone models sporadic Parkinson’s disease in *Drosophila melanogaster*. J. Neurosci. Off. J. Soc. Neurosci..

[72] Chaudhuri A., Bowling K., Funderburk C., Lawal H., Inamdar A., Wang Z., O’Donnell J.M. (2007). Interaction of genetic and environmental factors in a *Drosophila* parkinsonism model. J. Neurosci. Off. J. Soc. Neurosci..

[73] Meenakshi-Sundaram S., Mahadevan A., Taly A.B. (2008). Wilson’s disease: A clinico-neuropathological autopsy study. J. Clin. Neurosci. Off. J. Neurosurg. Soc. Australas..

[74] Guilarte T.R. (2010). Manganese and Parkinson’s disease: A critical review and new findings. Environ. Health Perspect..

[75] Barone M.C., Bohmann D. (2013). Assessing neurodegenerative phenotypes in *Drosophila* dopaminergic neurons by climbing assays and whole brain immunostaining. J. Vis. Exp..

[76] Chen A.Y., Wilburn P., Hao X., Tully T. (2014). Walking deficits and centrophobism in an α-synuclein fly model of Parkinson’s disease. Genes Brain Behav..

[77] Zhu Y., Lazopulo S., Syed S., Zhai R.G. (2022). Encyclopedia of Behavioral Neuroscience.

[78] Abdul K.S.M., Jayasinghe S.S., Chandana E.P., Jayasumana C., De Silva P.M.C. (2015). Arsenic and human health effects: A review. Environ. Toxicol. Pharmacol..

[79] MacDonald J.M., Beach M.G., Porpiglia E. (2006). The *Drosophila* cell corpse engulfment receptor Draper mediates glial clearance of severed axons. Neuron.

[80] Chiang A., Priya R., Ramaswami M., Vijayraghavan K., Rodrigues V. (2009). Neuronal activity and Wnt signaling act through Gsk3-beta to regulate axonal integrity in mature *Drosophila* olfactory sensory neurons. Development.

[81] Kazama H., Yaksi E., Wilson R.I. (2011). Cell death triggers olfactory circuit plasticity via glial signaling in *Drosophila*. J. Neurosci. Off. J. Soc. Neurosci..

[82] Hueston C.E., Olsen D., Li Q., Okuwa S., Peng B., Wu J., Volkan P.C. (2016). Chromatin Modulatory Proteins and Olfactory Receptor Signaling in the Refinement and Maintenance of Fruitless Expression in Olfactory Receptor Neurons. PLoS Biol..

[83] Muñiz-Ortiz J.G., Shang J., Catron B., Landero J., Caruso J.A., Cartwright I.L. (2011). A transgenic *Drosophila* model for Arsenic methylation suggests a metabolic rationale for differential dose-dependent toxicity endpoints. Toxicol Sci..

[84] Niño S.A., Chi-Ahumada E., Ortíz J., Zarazua S., Concha L., Jiménez-Capdeville M.E. (2020). Demyelination associated with chronic Arsenic exposure in Wistar rats. Toxicol. Appl. Pharmacol..

[85] Cabral Pinto M.M., Marinho-Reis A.P., Almeida A., Ordens C.M., Silva M.M., Freitas S., Simões M.R., Moreira P.I., Dinis P.A., Diniz M.L. (2018). Human predisposition to cognitive impairment and its relation with environmental exposure to potentially toxic elements. Environ. Geochem. Health.

[86] Yu C.C., Chang F.C., Hong Y.H., Li J.C., Chen P.L., Chen C.H., Chiu T.W., Lu T.T., Wang Y.M., Kao C.F. (2021). Assessing the cognitive status of *Drosophila* by the value-based feeding decision. NPJ Aging Mech. Dis..

[87] Mundey M.K., Roy M., Roy S., Awasthi M.K., Sharma R. (2013). Antioxidant potential of Ocimum sanctum in Arsenic-induced nervous tissue damage. Braz. J. Vet. Pathol..

[88] Tyler C.R., Allan A.M. (2014). The Effects of Arsenic Exposure on Neurological and Cognitive Dysfunction in Human and Rodent Studies: A Review. Curr. Environ. Health Rep..

[89] Chandravanshi L.P., Yadav R.S., Shukla R.K., Singh A., Sultana S., Pant A.B., Parmar D., Khanna V.K. (2014). Reversibility of changes in brain cholinergic receptors and acetylcholinesterase activity in rats following early life Arsenic exposure. Int. J. Dev. Neurosci. Off. J. Int. Soc. Dev. Neurosci..

[90] Aoyama K., Watabe M., Nakaki T. (2008). Regulation of neuronal glutathione synthesis. J. Pharmacol. Sci..

[91] Anushree A., Ali M.Z., Ahsan J. (2022). Acute Exposure to Arsenic Affects Cognition in *Drosophila melanogaster* Larvae. Entomol. Appl. Sci. Lett..

